# Phylodynamic analysis of HIV-1 subtypes B, C and CRF 02_AG in Senegal

**DOI:** 10.1016/j.epidem.2019.100376

**Published:** 2019-11-14

**Authors:** Fabrícia F. Nascimento, Stefan Baral, Lily Geidelberg, Christinah Mukandavire, Sheree R. Schwartz, Gnilane Turpin, Nguissali Turpin, Daouda Diouf, Nafissatou Leye Diouf, Karleen Coly, Coumba Toure Kane, Cheikh Ndour, Peter Vickerman, Marie-Claude Boily, Erik M. Volz

**Affiliations:** a Department of Infectious Disease Epidemiology, Imperial College London, Norfolk Place W2 1PG, UK; b Department of Epidemiology, Johns Hopkins School of Public Health, Baltimore, MD, USA; c Enda Sante, Dakar, Senegal; d Institut de Recherche en Santé, de Surveillance Epidemiologique et de Formations, Dakar, Senegal; e Division de La Lutte Contre Le Sida et Les IST, Ministry of Health, Dakar, Senegal; f Population Health Sciences, Bristol Medical School, University of Bristol, Bristol, UK; g MRC Centre for Global Infectious Disease Analysis, Imperial College London, UK

**Keywords:** HIV, Phylodynamics, Key populations, Coalescent

## Abstract

Surveillance of HIV epidemics in key populations and in developing countries is often challenging due to sparse, incomplete, or low-quality data. Analysis of HIV sequence data can provide an alternative source of information about epidemic history, population structure, and transmission patterns. To understand HIV-1 dynamics and transmission patterns in Senegal, we carried out model-based phylodynamic analyses using the structured-coalescent approach using HIV-1 sequence data from three different subgroups: reproductive aged males and females from the adult Senegalese population and men who have sex with other men (MSM). We fitted these phylodynamic analyses to time-scaled phylogenetic trees individually for subtypes C and CRF 02_AG, and for the combined data for subtypes B, C and CRF 02_AG. In general, the combined analysis showed a decreasing proportion of effective number of infections among all reproductive aged adults relative to MSM. However, we observed a nearly time-invariant distribution for subtype CRF 02_AG and an increasing trend for subtype C on the proportion of effective number of infections. The population attributable fraction also differed between analyses: subtype CRF 02_AG showed little contribution from MSM, while for subtype C and combined analyses this contribution was much higher. Despite observed differences, results suggested that the combination of high assortativity among MSM and the unmet HIV prevention and treatment needs represent a significant component of the HIV epidemic in Senegal.

## 1. Introduction

There are an estimated 36.7 million people around the world living with HIV with approximately 1.8 million newly infected in 2016 equating to approximately 5000 new infections per day (UNAIDS, 2017). Notably, 64% of incident HIV infections happen across sub-Saharan Africa (UNAIDS, 2017). In Senegal, 1100 new HIV infections have been estimated in 2016 among adults (UNAIDS, 2017) in which HIV prevalence is concentrated among key populations including men who have sex with other men (MSM).

Studies carried out among MSM in Senegal have showed stable and high HIV prevalence of 22.4% and 21.8%, including data from 2004 and 2007, respectively (Wade et al., 2005, 2010). A more recent study showed a prevalence in Senegal MSM population of 23.5% (Lyons et al., 2017). While generalizability of these estimates is limited by the number of sample locations, they do suggest a concentration of HIV in MSM which is further reflected by genetic populations structure of the virus. For example, these surveys have shown that in Senegal HIV-1 subtype C is the predominant subtype circulating among MSM, but HIV-1 CRF 02_AG predominates among the broader population of people living with HIV by a large margin (Ndiaye et al., 2009, 2013; Diop-Ndiaye et al., 2010), which shows a high degree of compartmentalization of these risk groups.

The high prevalence of subtype C among MSM may potentially lead to an increase of subtype C among other reproductive aged adults living with HIV if a substantial proportion of new HIV infections in women are acquired through sex with MSM. This is plausible because more than 90% of MSM in Senegal have reported having sex with women (Wade et al., 2005; Jung et al., 2012). In fact, prevalence of subtype C among reproductive aged adults living with HIV increased from 4% in 2000 to approximately 10% in 2010 (Jung et al., 2012), but the extent to which this is attributable to transmission by MSM to female partners has not been estimated.

To better understand HIV transmissions and the contribution of the unmet HIV prevention needs among MSM to the HIV epidemics in Senegal, we carried out a model-based phylodynamic analysis using HIV-1 genetic sequence data collected from both self-reported MSM and the general population. This analysis combined a phylogenetic component whereby a time-scaled phylogenetic tree was estimated from sequence data, with a model-fitting component whereby a semi-parametric compartmental epidemiological model was fitted to phylogenetic trees (Volz, 2012). The time-scaled phylogenetic tree is used to describe the evolutionary history of the pathogen and provides information on past transmission dynamics that is otherwise difficult to obtain using more traditional epidemiological methods. The mathematical model describes transmissions between the different groups of individuals, including MSM and reproductive aged men and women.

Recent advances in phylodynamic analysis methodology have enabled estimation of recent incidence trends and transmission patterns between risk groups (Volz and Siveroni, 2018; Volz et al., 2013). Largescale blinded simulation experiments have shown that model-based phylodynamic methods provide a viable strategy for estimating transmission patterns which are not identifiable from non-genetic surveillance data (Ratmann et al., 2016). In this paper, we employed model-based phylodynamic analysis to elucidate epidemiological trends for HIV-1 subtypes B, C, and CRF 02_AG circulating in Senegal. Similar analyses have previously provided valuable insights into transmission patterns in Nigeria (Volz et al., 2017a) and South Africa (Rasmussen et al., 2018).

## 2. Material and methods

### 2.1. Data

We retrieved from the Los Alamos HIV database (www.hiv.lanl.gov) 541 DNA sequences from partial HIV-1 *pol* gene, comprising protease and partial reverse transcriptase, from Senegal. These consisted of 39 sequences from subtype B, 107 from subtype C and 395 from subtype CRF 02_AG; and one sequence per patient.

We only retrieved DNA sequences with length greater than 1000 base pairs (bp). These sequence data were matched with associated epidemiological metadata on risk group, location of sampling, sex and collection date of HIV samples compiled at the Institut de Recherche en Santé, de Surveillance Epidémiologique et de Formation (IRESSEF), Dakar, Senegal.

In order to account for external introductions of HIV lineages into Senegal, we also retrieved additional sequences from outside Senegal by *blastn* (Altschul et al., 1990) using custom Python scripts (https://github.com/thednainus/senegalHIVmodel). Each Senegal sequence was compared to the nucleotide collection database (*nt*) which is a collection of sequences from the International Nucleotide Sequence Database Collaboration (INSDC). This comparison was carried out to obtain the best match for each sequence, which was only kept if the country of origin was known and it was not from Senegal. We only retrieved non-identical GenBank accession numbers that we referred to as “close global reference” (CGR) sequences. All CGR and Senegal sequences were analysed together and by subtype. The inclusion of CGR sequences allowed phylodynamic analyses to account for importation of lineages into Senegal (see section Phylodynamic Analyses).

For subtype B and C, three sequences from subtype D were used as an outgroup in the phylogenetic analysis (GenBank accession numbers: AY371157, AY253311 and K03454). Similarly, for subtype CRF 02_AG, three reference sequences from subtype A were used as outgroups (GenBank: AB253429, DQ676872 and AB253421). Outgroup sequences were used to root the phylogenetic trees by subtype (see section Molecular Clock Analysis).

DNA sequence alignment using only one sequence per patient was generated for each subtype with default settings using MUSCLE version 3.8.31 (Edgar, 2004) as implemented in ALIVIEW version 1.18.1 (Larsson, 2014). Alignments were further manually refined using the reference subtype B sequence HXB2 (GenBank: K03455) to align sequences by codon position. Columns of the alignment in which the majority of sequences were missing a nucleotide were removed (Yang, 2014). Multiple nucleotide positions on HIV-1 *pol* known to be under strong pressure to evolve antiretroviral drug resistance should not be included in phylogenetic analyses. Because of that, we masked in all alignments drug resistance sites using the function *seq.rm.drugresistance* from the R (version 3.4.3) package *big.phylo* version 1.0.0 (Ratmann, 2018).

This package uses the drug resistance mutation sites from the International Antiviral Society-USA (Wensing et al., 2015). Finally, to check for the presence of recombination within each alignment we used the *Phi* test (Bruen et al., 2006) as implemented in SPLITSTREE version 4.14.5 (Huson and Bryant, 2006). Recombination between HIV-1 subtypes were also further checked using the REGA HIV-1 subtyping tool version 3 (Pineda-Peña et al., 2013).

Including CGRs, a total of 617 sequences were analysed: 46 sequences from subtype B, 123 from subtype C, and 448 from subtype CRF 02_AG. These included 541 sequences from Senegal and 76 CGRs. The median age of Senegalese individuals analysed was 34 (IQR = 18–57) years old; 45% were females and 55% were males. From those, 91% were from Dakar and 9% from other cities in Senegal. Sample dates varied between 1990 and early 2014. The median year for all HIV-1 sample collection for subtype B was 2004 (IQR = 1999–2007), for subtype C was 2007 (IQR = 2004–2008) and for subtype CRF 02_AG was 2008 (IQR = 2003–2010).

### 2.2. Phylogenetic analysis

DNA sequence alignments were used for phylogenetic reconstruction by maximum likelihood (ML) using RAXML-NG version 0.5.1 (Kozlov et al., 2018). We independently reconstructed phylogenetic trees for each subtype and using 4 different DNA substitution models: (1) GTR + Γ (General Time Reversible (Tavaré, 1986; Zharkikh, 1994; Yang, 1994) plus gamma distribution (Yang, 1996) with 4 categories); (2) GTR + I + Γ (where I is the proportion of invariable sites); (3) GTR + R (where R is the FreeRate model (Soubrier et al., 2012)); and (4) GTR + Γ and using 2 partitions for the data, one partition for first and second codon positions, and another partition for the third codon position. We also used the parsimony-based randomized stepwise addition trees as starting trees to search for the best ML tree.

We implemented all analyses using the computing resources of the Open Science Grid (OSG) (Pordes et al., 2007; Sfiligoi et al., 2009). To estimate the ML tree, we ran 20 jobs per alignment per DNA substitution model in parallel. We used parsimony starting trees to estimate the ML tree. We chose the tree with the highest likelihood as the best ML tree. Similarly, to calculate branch support for each tree, we ran 1000 independent bootstrapped trees per alignment in parallel which were merged into a single file to calculate the bootstrap support for each branch of the best ML tree.

The best DNA substitution model was selected using the Bayesian Information Criterion (BIC). All subsequent analyses were carried out using the best ML tree as chosen by BIC.

### 2.3. Molecular clock analysis

Estimated phylogenetic trees have branch lengths in nucleotide substitutions per site. To convert these branch lengths in unit of calendar time, we fit a relaxed molecular clock to the estimated ML tree using the function *dater,* with default options, from the R package *treedater* version 1.0 (Volz and Frost, 2017). For these analyses, we rooted the trees using outgroup sequences, which were dropped before fitting the relaxed clock. A wide range of sample dates between 1990 and 2014 enabled precise estimation of molecular clock rates. Tree manipulations were carried out using the R package *ape* version 5.2 (Paradis et al., 2004). We also provided uncertainty bounds for samples with uncertainty in their collection date, for example, samples in which only year was provided. We used these dated phylogenetic trees in all phylodynamic analyses. The same relaxed clock model was fitted to bootstrap trees without temporal constrains to reduce computational time.

### 2.4. Phylodynamic analysis

Phylodynamic analyses were carried out using a structured coalescent model (SCM) (Volz, 2012) which conditions on different sampling dates in different risk groups. SCM uses a dated phylogenetic tree to describe the transmission history of HIV between individuals, and a mathematical model to describe the transmission patterns. It also assumes that each sequence is associated to metadata – traits associated to each individual. In our analyses these traits were from one of the following groups: *gpf* (presumed heterosexual reproductive aged women); *gpm* (presumed heterosexual reproductive aged men); *msm* (men who have sex with other men); and *src* (source), which represented the global reservoir of HIV which donates lineages to Senegal. Source sequences were represented by the CGR sequences which were sequences from other countries and not from Senegal. The inclusion of these sequences allows the model to account for importation of lineages into Senegal.

Phylodynamic analyses were individually carried out using phylogenetic trees for subtype C, subtype CRF 02_AG, and the combined tree which comprised merging as a polytomy the dated trees for subtypes B, C and CRF 02_AG. We also removed from the trees samples for which risk group or sex was not available and samples collected from children. For analyses by subtype, we used all sequences from Senegal, while for the combined tree, we used only sequences from the capital city of Dakar. The latter was preferred to reduce computational time and assume a more homogeneous population for better population estimates.

We used ordinary differential equations (ODEs) to understand the dynamics of HIV infections and transmission rates. We based our ODEs on a compartmental infectious disease model with 4 compartments representing the number of infected individuals in *gpf, gpm* and *msm* risk groups. We also modelled importation of HIV lineages to *gpf, gpm* and *msm* by adding an additional compartment referred to as *src*. We modelled the *src* compartment as having a constant effective population size with two parameters: the effective source population size and the importation rate. The choice of a constant size was motivated by a desire to keep the number of free parameters down. Realism of the source compartment is relatively unimportant, since in the coalescent model it serves merely as a reservoir for lineages which have a common ancestor in the distant past predating epidemic expansion in Senegal.

The ODEs representing our mathematical model were based on one stage of HIV infection: people living with HIV would not recover from the infection and would cease to transmit at a rate γ per person per year. We used only one stage of HIV infection because the available metadata did not have information that could be used to determine the stage of HIV infection at the time of sample collection.

Incidence of HIV infection was parameterized using a flexible piecewise linear function which depended only on time and the current number of infectious individuals. Note that these models do not make use of the *common mass action* assumption whereby incidence increases proportionally to the product of infectious and susceptible individuals (Anderson and May, 1991). For this reason, we did not need to model the number of susceptible individuals through time. The per-capita transmission rates (units of transmissions per person per unit time) are denoted by μ(t) and λ(t), respectively in *gp* (all reproductive aged adults) and *msm.* The functions μ(t) and λ(t) were piecewise linear functions with 4 parameters each; 3 parameters for transmission rates and 1 parameter for the interval (time). The reproduction number, $R_{0}(t)$, was computed as μ(t)/γand λ(t)/γ for *gp* and *msm*.

The models also included parameters to control relative transmission rates between different risk groups. Transmissions by *msm* can infect susceptible hosts in *gpf* or *msm*, and we used the parameter q∈(0,1) to represent the probability that a transmission by *msm* will infect *msm* and with probability 1−q will infect *gpf*. Similarly, p∈(0,1) represents the probability that a transmission by *gpf* will infect *gpm* and with probability 1−p such a transmission will infect *msm*. Finally, whereas women may have higher susceptibility to infection during heterosexual intercourse (Boily et al., 2009; Patel et al., 2014), the model accounts for asymmetric risk of infection between men and women, and the parameter ψ>0 represents the transmission risk ratio of males relative to females.

The ODEs, as explained above, describing the effective number of people living with HIV *gpf* (*x*), *gpm* (*y*) and *msm* (*z*) throughout time are represented below, and for a summary of each parameter in the equations see Table 1:

(1) $$\dot{x}=\psi \mu (t)y+(1-q)\lambda (t)z-\gamma x$$

(2) $$\dot{y}=p\mu (t)x-\gamma y$$

(3) $$\dot{z}=(1-p)\mu (t)x+q\lambda (t)z-\gamma z$$

We model movement of lineages between Senegal and *src* as a migration process that depends the current number infected in each deme. The rate of lineages migrating from *src* to, for example, *msm*, is *υz*. The migration process is bidirectional and equal in magnitude, so migration from *msm* to *src* is given by the same rate. Consequently, this process has no influence over the size of *gpm,gpf,* or *msm* through time. It does, however, play a large role in computing the estimated ancestral probabilities for lineages being in the *src* deme.

In these phylodynamic analyses, we associated each tip of the dated phylogenetic tree a trait based on available metadata, that in our model were *gpf, gpm, msm* and *src.* These represent self-reported classification of risk group. However, same-sex practices are illegal in Senegal, and there is significant stigma affecting MSM (Lyons et al., 2017). It is then possible that self-reported *gpm* are in fact *msm.* Based on this idea, we fit three variations of the model for the analyses with the combined tree (1 to 3; see below) and two variations of the model (1 and 2; see below) for the analyses by subtype because of limited amount of sequences.

1. We assigned each sequence to its respective risk-group in the phylogenetic tree a value of 100% in the respective self-reported risk group;
2. We assumed some uncertainty in the self-reported *gpm* by arbitrarily assigning to every *gpm* sequence a value of 50% of being *gpm* and 50% of being *msm*;
3. We removed all *gpm* sequences from the phylogenetic tree.

To each of these enumerated variations, we also adapted the model likelihood to include a term based on previously published HIV prevalence in *gpm* and *msm* in Dakar, Senegal (Mukandavire et al., 2018). For further information on how likelihoods were calculated see Supplementary Material. In summary, we fit six variations of the mathematical model for the combined tree, and four variations of the mathematical model for the phylodynamic analyses by subtype. We used as base model, the model using self-reported risk group and surveillance data in the calculation of the likelihood. The other variations of the mathematical model were used as sensitivity analyses to understand to what extent the uncertainty of the number of *gpm* would affect the estimated results.

Model variations were fitted using the R package *phydynR* version 0.1 (Volz, 2017). The structured coalescent likelihood was computed using the *QL* approximation described in Volz and Siveroni (2018). Model parameters were estimated by differential evolution Markov chain Monte Carlo (MCMC) *zs* sampler (ter Braak and Vrugt, 2008) using the development version of the R package *BayesianTools* version 0.1.5 (Hartig et al., 2018). All analyses were carried out in parallel using the computing resources of the OSG (Pordes et al., 2007; Sfiligoi et al., 2009). For more details see Supplementary Material.

After estimating the parameter of our mathematical model, we derived for each group (*gpf, gpm* and *msm*) the effective number of infections and the population attributable fraction (PAF) through time from simulations based on the MCMC posterior distributions. The effective number of infections was defined as the number of infected hosts within the structured coalescent model fitted to the virus phylogeny. These population sizes yield a distribution of branch lengths which are consistent with observations from the virus phylogeny. The effective population size is not the same but is approximately proportional as the number of infected hosts. Note that any unmodeled factor which influences evolution or transmission of the pathogen can influence the relationship between effective and true population sizes, including within-host evolution (Volz et al., 2017b), variance in transmission rates between individuals, and major epidemiological phenomena, such as the roll-out of HAART (highly active antiretroviral therapy) in the 21st century Volz et al. (2013). PAF through time was defined as the probability that an infection at time t was generated by a given risk group, represented by *gpf, gpm* and *msm*. Simulations for phylodynamic analyses were carried out from 1978 to 2014 (see Supplementary Material for more information).

In the main text, we only reported results for PAF and effective number of infections derived from the base model: phylodynamic analysis carried out using self-reported risk group and adding a term based on previously published HIV prevalence in *gpm* and *msm* in Dakar, Senegal (see Supplementary Material). Also, there are no statistical analyses that we could use to compare the best phylodynamic analyses to derive PAF and effective number of infections from the mathematical models we tested. We therefore chose a base model based on self-reported risk groups and surveillance data and carried out sensitivity analyses using variations of the mathematical model. We also aimed to compare our MSM PAF results to the 1-year PAF reported in Mukandavire et al. (2018). Note that these authors only reported 1-year MSM PAF for 1995, 2005 and 2015. They also did not report the effective number of infections or 1-year PAF for heterosexual reproductive aged females and males.

### 2.5. Code and data availability

We have uploaded all Python and R scripts used in our analysis as a research compendium in GitHub (https://github.com/thednainus/senegalHIVmodel). DNA multiple sequence alignments and phylogenetic trees used in phylodynamic analyses can also be found in this same research compendium in GitHub.

## 3. Results

No evidence of recombination was detected for any of the three subtypes using the *Phi* test, and phylogenetic trees were then individually reconstructed for each subtype. For all subtypes, partitioning the data by codon position generated an ML tree with the lowest BIC. This is not surprising given results on best substitution models for protein-coding sequences (Shapiro et al., 2006). These trees with the lowest BIC were used in all subsequent analyses. Estimated rates of molecular clock evolution were highly consistent across subtypes. These rates were 0.0022, 0.0019, and 0.0021 substitutions per site per year for 02_AG, B and C respectively. A relaxed clock was strongly supported in all cases with a coefficient of variation of rates of 0.30–0.38.

### 3.1. Phylodynamic analyses

Phylodynamic analyses were carried out for three different phylogenetic trees: subtype C, subtype CRF 02_AG and for the combined tree (including sequences from subtypes B, C and CRF 02_AG). We did not analyse subtype B phylodynamics because of the limited number of available sequences.

For the combined tree, phylodynamic analyses were carried out for a total of 463 sequences: 387 from Dakar and 76 CGRs. This combined tree comprised 267 sequences from subtype CRF 02_AG, 90 sequences from subtype C and 30 sequences from subtype B. We also individually analysed the phylodynamics for 355 (302 from Senegal) sequences from subtypes CRF 02_AG and 112 (96 from Senegal) sequences from subtype C.

For the combined analyses, a higher proportion of *msm* was observed within subtype C, while the highest proportion of *gpf* was observed within subtype CRF 02_AG (Table 2). For subtype C analyses, 41.9% were from the general population, 43.8% were from *msm*, and 14.3% represented *source*. For CRF 02_AG analyses, 73.5% were from the general population, 11.6% from *msm* and 14.9% represented *source.*

### 3.2. Effective number of infections

**Fig. 1.**
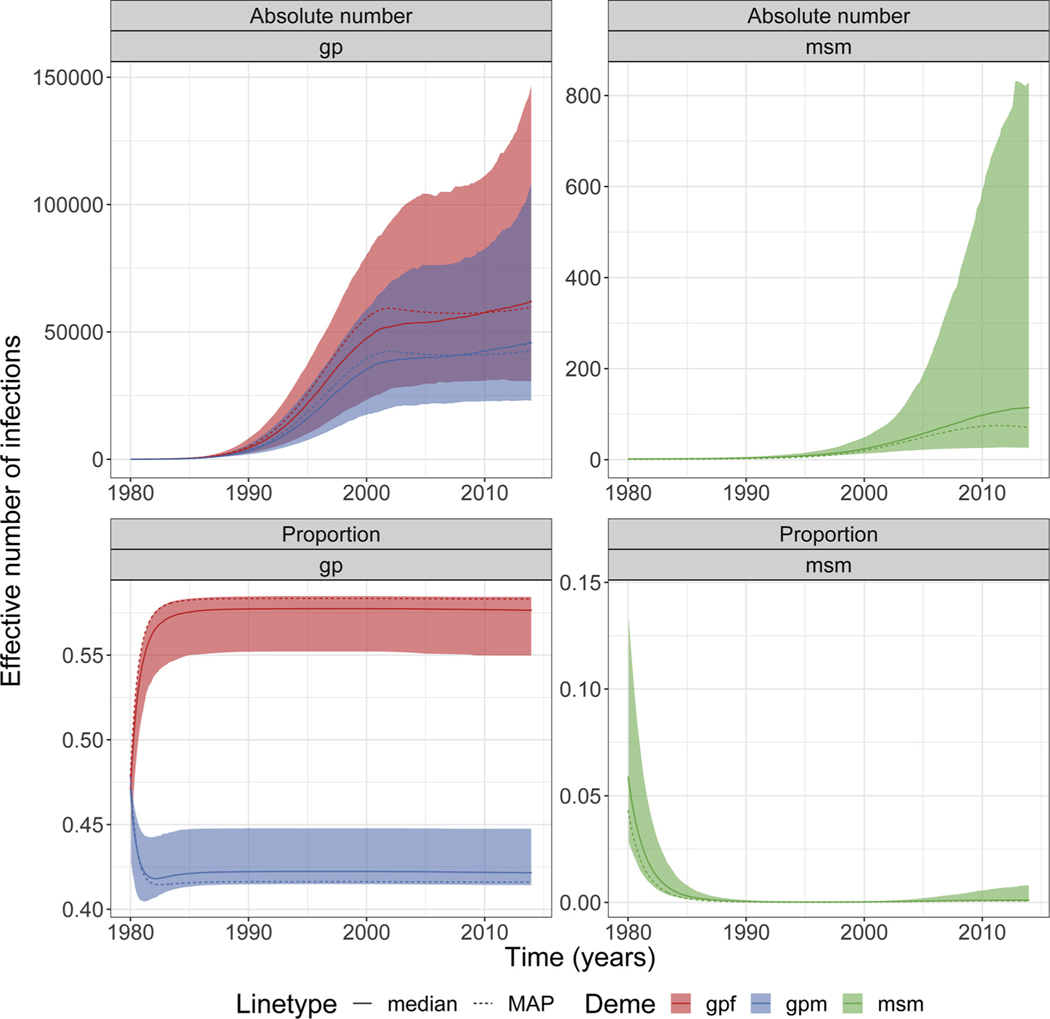
Effective number/proportion of infections in risk groups for subtype CRF 02_AG. Plots showing the absolute number and proportion of the effective number of infections in each deme/group (*gpf, gpm* and *msm*) for the individual analyses for subtype CRF 02_AG. Shaded area represents the 95% credible interval. MAP = maximum a posteriori.

For subtype CRF 02_AG, we estimated very small numbers of infected *msm* with little variation through time when comparing to the other reproductive aged adults (Fig. 1). Consequently the proportion of infections in *gpf, gpm* and *msm* are approximately constant through time neglecting transient effects during the very early HIV epidemic. The proportion of infected individuals was higher for *gpf* than *gpm* due to asymmetric infection risk (Fig. 1). The absolute number of infections in *gpf, gpm* and *msm* increased with time and a much higher number of infected individuals were observed among all reproductive aged adults (Fig. 1), and that is reflected on the very small proportion of infected individuals in *msm.*

**Fig. 2.**
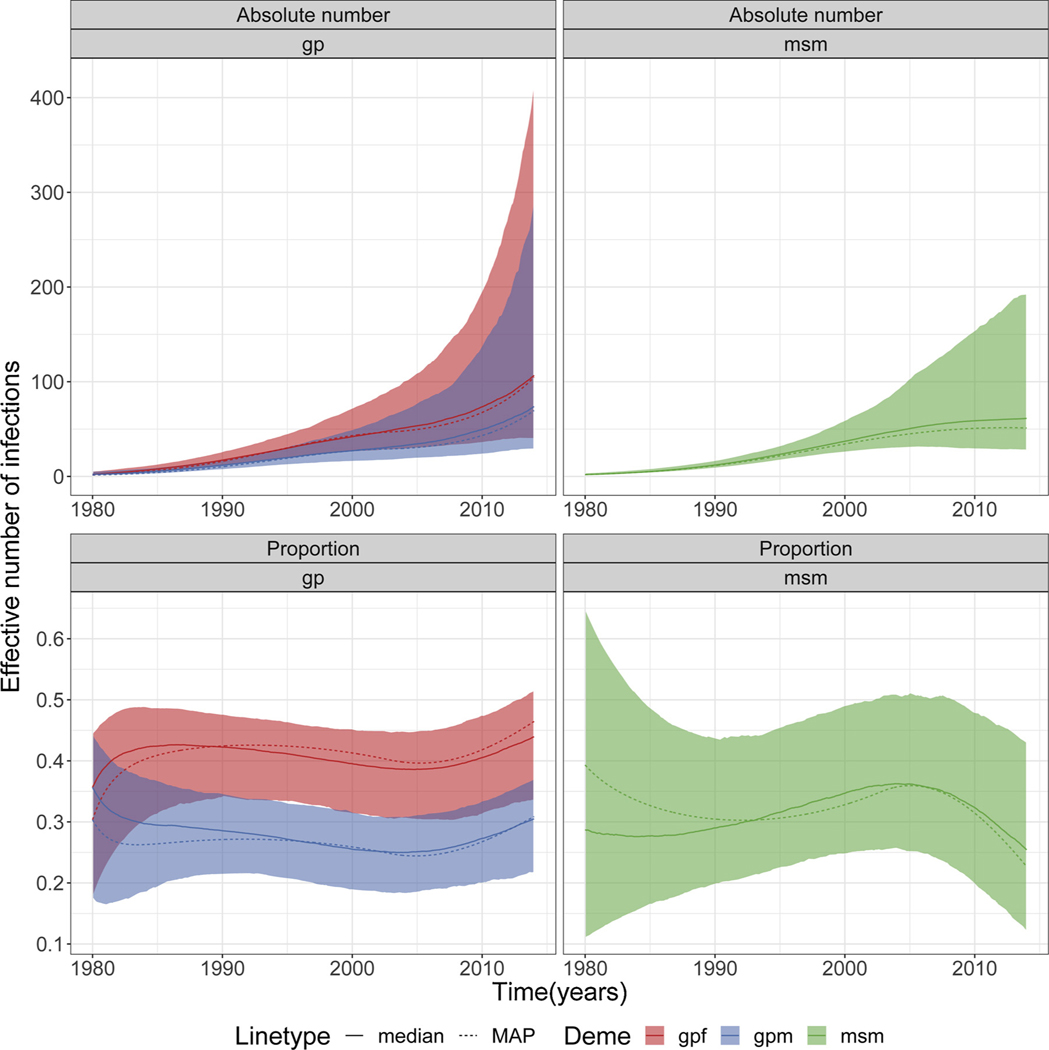
Effective number/proportion of infections in risk groups for subtype C. Plots showing the absolute number and proportion of the effective number of infections in each deme/group (*gpf, gpm* and *msm*) for the individual analyses for subtype C. Shaded area represents the 95% credible interval. MAP = maximum a posteriori.

We estimated that subtype C is highly concentrated in *msm* when comparing to subtype CRF 02_AG (Figs. 1 and 2). For *msm*, the proportion of infected individuals slightly increased with time, decreasing slightly at around 2005; while the absolute number gradually increased with time. While the absolute number gradually increased with time for both *gpf* and *gpm*, the proportions of infected individuals increased and decreased more sharply for *gpf* and *gpm*, respectively. We observed, in general, a higher proportion of infected *msm* individuals (including credible interval) than *gpf* and *gpm* (Fig. 2). In general, a higher proportion of infected individuals was observed for *gpf* than *gpm*.

**Fig. 3.**
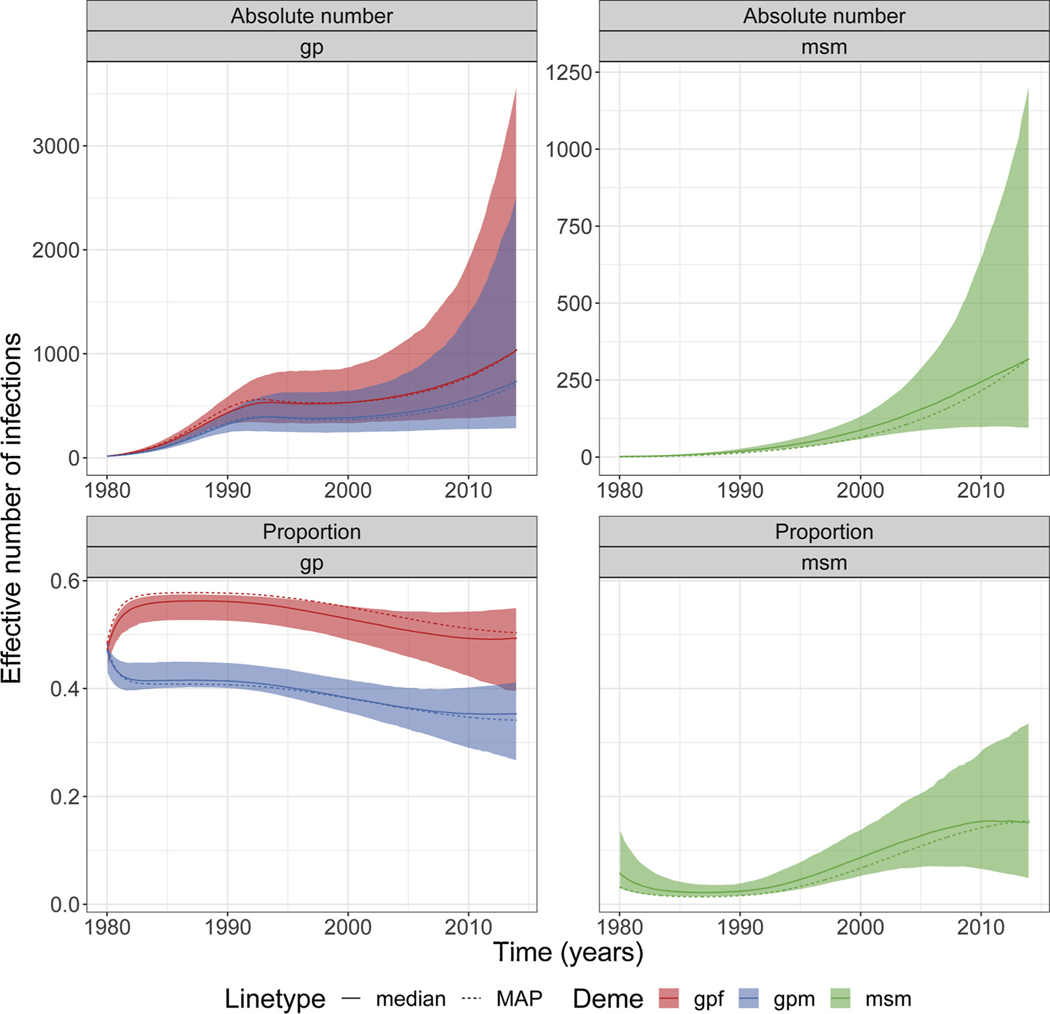
Effective number/proportion of infections in risk groups for the combined analyses. Plots showing the absolute number and proportion of the effective number of infections in each deme/group (*gpf, gpm* and *msm*) for the combined analyses (including subtypes B, C and CRF 02_AG). Shaded area represents the 95% credible interval. The *y*-axis for proportion of infections in *msm* is the same as for *gp*. MAP = maximum a posteriori.

For the combined-subtype analyses, the proportion of infected *gpf* increased up to early 1980’s and soon after decreased with time (Fig. 3). For *gpm*, the proportion of infected individuals decreased over time (Fig. 3). In contrast, the proportion of infected *msm* increased with time and by 2014 were higher but similar to those observed in *gpm* (including credible interval; Fig. 3 and Table 3). While we observed a high number of infected individuals in *msm*, these absolute numbers were much higher (including credible interval) for the general population (Fig. 3; Table 3).

When comparing the proportion of effective number of infections in 2014, we observed a higher *gpf* proportion across all analyses (Table 3). We also observed a very low proportion of effective number of infections for *msm* when analysing subtype CRF 02_AG (Table 3).

For plots showing the proportion of effective number of infections derived from phylodynamic analyses carried out using the other variations of the mathematical model see Supplementary Material.

### 3.3. Population attributable fraction

**Fig. 4.**
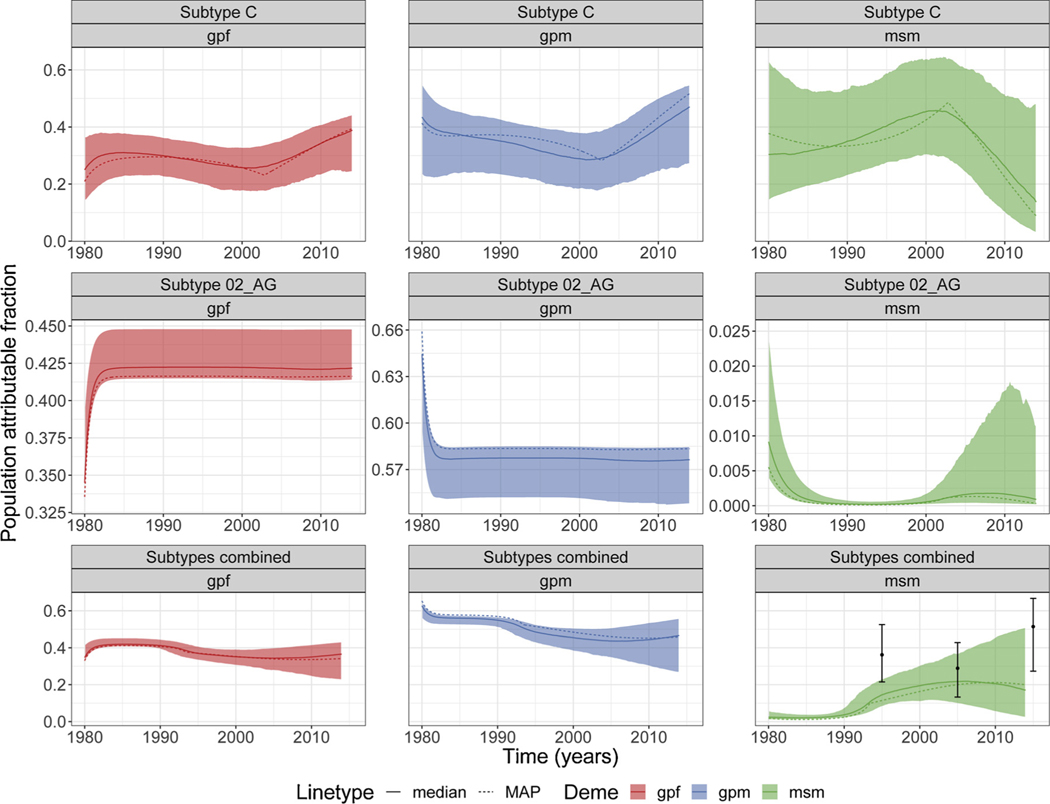
Population attributable fraction. Plots showing the population attributable fraction for each deme/group (*gpf, gpm* and *msm*) for the individual analyses for subtypes C and 02_AG, and for the combined analyses (including subtypes B, C and 02_AG). Point estimates and error bars in the last plot represents 1-year PAF estimated for MSM in Mukandavire et al. (2018). Shaded area represents the 95% credible interval. When not shown, *y*-axis is horizontally shared between plots. MAP =maximum a posteriori.

Subtype CRF 02_AG had very little contribution from *msm* and, consequently, the PAF for *gpf* and *gpm* reach equilibrium during epidemic expansion in the 1980’s. In 2014, PAF for *gpm* was about 16% higher than for *gpf,* while PAF for *msm* was very low (Fig. 4).

For subtype C, the PAF for both *gpf* and *gpm* decreased with time and around early 2000 started to increase. In general, *gpf* PAF was lower than *gpm*. In contrast, the PAF for *msm* increased with time reaching higher values (including credible interval) than *gpf* and *gpm*, and around early 2000 *msm* PAF started to decrease (Fig. 4).

For the combined analyses, the PAF for both *gpf* and *gpm* decreased with time, and the PAF for *gpm* was higher than *gpf*. In contrast, PAF for *msm* increased with time. By 2014, the PAF for *msm* was high and similar to that observed in the general population (Fig. 4; Table 3).

When comparing our most recent estimates for PAF in 2014, we observed that *gpf* PAF for subtype CRF 02_AG (0.42; 95% Credible Interval (95% CI): 0.41–0.45) were similar to estimates for subtype C (0.39; 95% CI: 0.24–0.44) and combined analyses (0.37; 95% CI: 0.23–0.43) (Table 3). PAF for *gpm* was the highest for subtype CRF 02_AG (0.58; 95% CI: 0.55–0.58) and very similar between subtype C (0.47; 95% CI: 0.27–0.55) and combined analyses (0.47; 95% CI: 0.27–0.56) (Table 3). On the other hand, *msm* PAF was very low for subtype CRF 02_AG (9 × 10^−4^; 95% CI: 1.1 × 10^−4^–0.01) and similar between subtype C (0.14; 95% CI: 0.03–0.48) and combined analyses (0.17; 95% CI: 0.02–0.51) (Table 3).

**Fig. 5.**
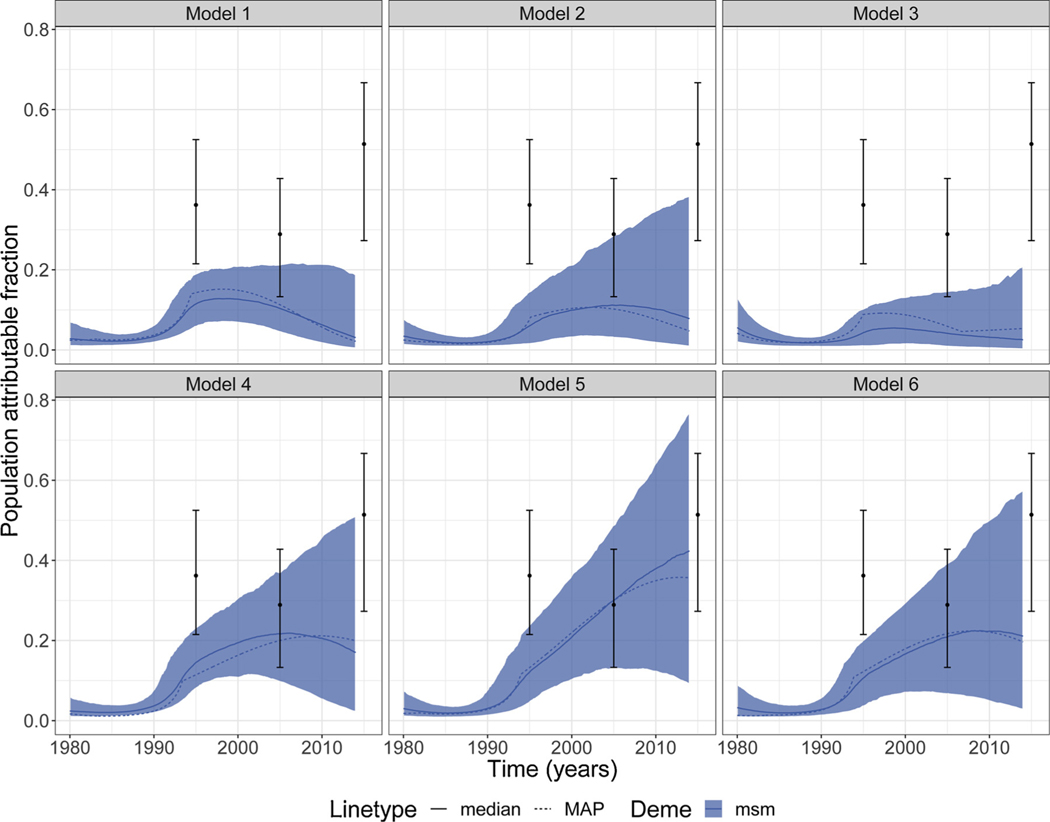
Sensitivity analyses. Plots showing the population attributable fraction for *msm* for the combined analyses (including subtypes B, C and 02_AG). Point estimates and error bars represent 1-year MSM PAF estimated in Mukandavire et al. (2018) for 1995, 2005 and 2015. Shaded area represents the 95% credible interval. MAP = maximum a posteriori.

For PAF derived from phylodynamic analyses carried out using the other variations of the mathematical model, see Supplementary Material. Note that, usually, when the prevalence term was not added to the calculation of the likelihood, 1-year MSM PAF for 1995, 2005 and 2015 reported by Mukandavire et al. (2018) did not coincide with our estimates (see Fig. 5 and Supplementary Material).

### 3.4. Transmission patterns

A small but significant net flow of transmissions was estimated from MSM to females which may be a consequence of higher prevalence among MSM and compounded by un-met prevention and treatment needs among MSM. In the combined-subtype analyses, 3.2% (95% CI: 0.6%–8.8%) of infections in heterosexual females were acquired from MSM, and 0.3% (95% CI: 0.1%–0.5%) in MSM acquired from heterosexual females (Table 4). For analyses using only subtype CRF 02_AG, a small proportion of transmissions were observed between MSM and heterosexual females and vice versa; while for analyses using only subtype C we estimated that approximately 2.5% (95% CI: 0.5%–8.6%) of infections were acquired by heterosexual females from MSM, and approximately 2.6% (95% CI: 0.9%–5.8%) of infections were acquired by MSM from heterosexual females.

### 3.5. Parameter estimates

Based on the incidence rates among all reproductive aged adults [μ(t)] and for MSM [λ(t)] we can estimate the basic reproduction number, $R_{0}$ (see Section 2). Our most recent estimates for 2014 showed a high $R_{0}$ for the reproductive aged adults when analysing subtype C (median: $R_{0}=1.59$) and the combined analyses (median: $R_{0}=1.29$). An $R_{0}$ less than 1.0 was observed for subtype CRF 02_AG reflecting decreasing epidemic prevalence within this subtype. On the other hand, $R_{0}$was higher for MSM for the combined analyses (median: $R_{0}=1.96$), and around 1.0 for subtypes C and CRF 02_AG (Table 5).

The probability of an infected *gpf* to infect a *gpm* was high and between 93% and 100% across analyses (Table 5). Similarly, the probability of an HIV-infected *msm* to infect another *msm* was also high and between 80% and 86% across analyses (Table 5). Our results also suggest that the probability (1−q) an MSM will infect a *gpf* is between 14% and 20% across analyses.

### 3.6. Sensitivity analyses

To understand how sensitive the mathematical model was to the number of self-reported *gpm* in the phylogenetic tree, we tested variations of the mathematical model (see Section 2). Fig. 5 shows *msm* PAF for Model 1 to Model 6. Model 1 to Model 3 were carried out using only genetic information while Model 4 to Model 6 were carried out using genetic information and surveillance data (see Section 2 and Supplementary Material). We also added 1-year MSM PAF for 1995, 2005 and 2015 reported by Mukandavire et al. (2018) for comparisons.

Our base model was Model 4 using information on self-reported risk groups and surveillance data. For the base model, we observed lower median estimates for 1995, 2005 and 2015 and smaller uncertainty range for 2005 and 2015 estimates when comparing to results reported by Mukandavire et al. (2018) (Fig. 5). For Model 5, estimates reported by Mukandavire et al. (2018) were within our credible interval for 2005 and 2015 estimates, but not for the 1995 estimate. Our Model 4 and Model 6 showed very similar estimates (Fig. 5). When only genetic data were considered (Models 1 to 3), our estimates were, in general, much lower than the ones reported by Mukandavire et al. (2018) (Fig. 5).

## 4. Discussion

This analysis provides additional insights into HIV epidemiological dynamics in Senegal using model-based phylodynamic methods applied to hundreds of HIV sequences. We observed substantial differences when comparing analyses based on combined HIV-1 subtypes (B, C and CRF 02_AG) to those analyses by particular subtypes (C or CRF 02_AG). For example, the proportion of effective infections were higher among MSM when analysing subtype C than in analyses of subtype CRF 02_AG. Trends observed for the proportion of effective number of infections among reproductive aged adults also differ. While the overall pattern for the combined analysis showed a decreasing proportion of infections among all reproductive aged adults relative to MSM, we observe a nearly time-invariant distribution for subtype CRF 02_AG and an increasing trend for subtype C.

A similar result for MSM PAF consistent with a previous epidemiological modelling study (Mukandavire et al., 2018) was estimated in one of our sensitivity analyses when we removed all self-reported general population males from our phylogenetic tree and added the prevalence term to the calculation of the likelihood (Model 5; Fig. 5). The estimates of 1-year PAF reported in Mukandavire et al. (2018) were in accordance to our estimates for 2005 and 2015 for Model 5 (Fig. 5), but our estimates were lower in 1995 when we estimated that the epidemic was expanding among MSM. Estimates reported by Mukandavire et al. (2018), usually, did not coincide with our MSM PAF estimates when our analyses were carried out without adding the prevalence term to the calculation of the likelihood (see Supplementary Material) (Fig. 5) suggesting that inclusion of non-genetic surveillance data is important to achieving stable estimates with the phylodynamic model.

Some differences observed between our analyses and Mukandavire et al. (2018) could be attributable to the different type of modelling and additional sources of surveillance data used. In Mukandavire et al. (2018), the authors used a dynamic HIV transmission model to investigate how the unmet HIV prevention and treatment needs among MSM, female sex workers (FSW) and their clients contributed to the overall HIV epidemics in Dakar, Senegal. Their model had more complex population structure involving the movement of individuals in six different sub-populations, stage of HIV infections and disease progression, and sex interactions which could result in HIV transmissions. Our model was more simple and focused on the transmission between three sub-populations (*gpf*, *gpm* and *msm*), but did include a more flexible function for the force of infection through time. Contrary to Mukandavire et al. (2018), we primarily used genetic data to infer the parameters of our model whereas Mukandavire et al. (2018) drew on a greater variety of previously published parameter estimates and epidemiological surveillance data over time. The scale of the epidemic size estimated by population genetic modeling is highly sensitive to unmodeled geographic structure and unmodeled variables that influence variance in transmission rates between individuals. It is currently infeasible to build and fit models that can adjust for all of these variables without extensive additional data. Since the primary aim of this analysis was to examine the epidemiological role of *msm*, we have remained agnostic regarding correspondence of our size estimates to the actual numbers of PLWHIV.

We tested six variations of the phylodynamic analyses with combined-subtype data and four variations of the phylodynamic analyses when analysing specific subtypes (see Section 2). We observed that results are sensitive to which data were included and assumptions made about metadata (Fig. 5). This sensitivity to model selection and data inclusion could be due to systematic differences in how sampling was conducted. Surveys which furnished HIV-1 sequence samples used respondent-driven and convenience samples for MSM but not the overall population (Wade et al., 2005, 2010; Lyons et al., 2017), and this sampling strategy was preferred due to MSM being highly stigmatised in Senegal (Lyons et al., 2017).

Our results may facilitate design of public health interventions as it supports the hypothesis that scaling up prevention efforts among key populations including MSM may generate a greater reduction in new infections. According to our results, the probability that an MSM would transmit HIV to a female sex partner is 14% to 20% (1−q; which is the probability an *msm* would transmit to a *gpf* in our mathematical model) across analyses (Table 4), consistent with previous reports that 90% of MSM have female sex partners in Senegal (Wade et al., 2005; Jung et al., 2012). We also estimate that a small but disproportionately higher number of transmissions among women are acquired from MSM, than vice versa (Table 4). Similar transmission patterns between MSM and females were recently reported in Nigeria, also using phylodynamic methods (Volz et al., 2017a).

One limitation of these models is that we are unable to select the best model and/or analyses to draw our final conclusions on HIV transmission in Senegal because there is no statistical method available to carry out such model comparison. We can clearly show a difference between the individual analyses by subtypes C and CRF 02_AG, which can be a consequence of the sampling strategy used to collect these data. We also did not include female sex workers in our model because there were no sequence data available from these groups.

We also lacked metadata such as CD4 cell counts and HIV incidence assays which would be useful for controlling for differential transmission rates over the course of infection. Future studies could resolve these ambiguous results by conducting large randomized sampling of MSM, other core groups, and the general population combined with sequencing and collection of clinical data that is informative about time since infection. Moving forward necessitates the safe and systematic collection of risk status data for people living with HIV in Senegal, including FSW and their clients, which can be achieved in the context of passive case-based surveillance or in household surveys. Currently, the criminalization of same-sex practices likely limits disclosure and ultimately affects our ability to fully ascertain HIV transmission dynamics in Senegal.

Taken together, these analyses highlight the disproportionate burden of HIV among MSM in West Africa. These conclusions, while tentative, support the case for increased coverage of HIV prevention and treatment services for MSM with evidence-based and rights-affirming interventions.

## Supplementary Material

1

## Figures and Tables

**Table 1:** Summary of dynamic variables and parameters in the mathematical model.

Variable or parameter	Definition

x(t)	Number of infected *gpf*
y(t)	Number of infected *gpm*
z(t)	Number of infected *msm*
ψ	Risk ratio of *gpm* to transmit to a *gpf*
p	Probability of *gpf* to transmit to a *gpm*
q	Probability of *msm* to transmit to another *msm*
γ	Removal rate
μ(t)	Piecewise linear function for per-capita transmission rate in *gp*
λ(t)	Piecewise linear function for per-capita transmission rate in *msm*

**Table 2:** Distribution of HIV-1 in Dakar by subtype and self-reported risk-group (discrete-trait) used in the combined analyses. Numbers in brackets are proportions.

	02_AG	B	C

*gpf*	153 (0.57)	7 (0.23)	22 (0.24)
*gpm*	87 (0.33)	9 (0.30)	24 (0.27)
*msm*	27 (0.10)	14 (0.47)	44 (0.49)
Total	267	30	90

**Table 3:** Estimates in 2014. Estimates (median and 95% credible interval) for the proportion of the effective number of infections (*x*, *y* and *z*) and PAF (for *gpf*, *gpm* and *msm*) by subgroups for subtype CRF 02_AG, subtype C and combined analyses.

	Subtype 02_AG	Subtype C	Subtypes combined

*x* (*gpf*)	0.58 (0.55–0.59)	0.44 (0.34–0.52)	0.49 (0.39–0.55)
*y* (*gpm*)	0.42 (0.41–0.45)	0.31 (0.22–0.37)	0.35 (0.27–0.41)
*z* (*msm*)	0.001 (2 × 10^−4^–0.008)	0.25 (0.12–0.43)	0.15 (0.05–0.34)
PAF (*gpf*)	0.42 (0.41–0.45)	0.39 (0.24–0.44)	0.37 (0.23–0.43)
PAF (*gpm*)	0.58 (0.55–0.58)	0.47 (0.27–0.55)	0.47 (0.27–0.56)
PAF (*msm*)	9 ×10^−4^ (1.1 × 10^−4^–0.01)	0.14 (0.03–0.48)	0.17 (0.02–0.51)

**Table 4:** Transmissions. Proportion of infections from msm to gpf and from gpf to msm for subtype CRF 02_AG, subtype C and subtypes combined (including subtypes B, C and CRF 02_AG) from 1978 to 2014.

Transmissions	Subtype 02_AG	Subtype C	Subtypes combined

From *msm* to *gpm*	< 0.01 (< 0.01–0.01)	0.025 (0.005–0.086)	0.032 (0.006–0.088)
From *gpf* to *msm*	< 0.01 (< 0.01– < 0.01)	0.026 (0.009–0.058)	0.003 (0.001–0.005)

**Table 5:** Estimates of parameter of interest. Estimates (median and 95% credible interval) for the basic reproduction number ($R_{0}$), the probability an infected *gpf* would infect a *gpm* (p) and the probability an infected *msm* would infected another *msm* (q) for subtype CRF 02_AG, subtype C and combined analyses. $R_{0}$was calculated for 2014 and p and q was calculated for a period between 1978 and 2014.

	Subtype 02_AG	Subtype C	Subtypes combined

$R_{0}$(gp)	0.87 (0.53–1.80)	1.59 (0.67–2.91)	1.29 (0.73–2.06)
$R_{0}$(msm)	1.05 (0.52–2.82)	1.00 (0.54–2.13)	1.96 (0.77–3.41)
p	1.00 (1.00–1.00)	0.93 (0.85–0.98)	0.99 (0.99–1.00)
q	0.86 (0.73–0.95)	0.82 (0.72–0.89)	0.80 (0.67–0.88)
