# Supplementary material for: Phylodynamic analysis of HIV-1 subtypes B, C and CRF 02_AG in Senegal
Source: Epidemics. Author manuscript; Available in PMC 2023 Apr 1. (PMC10066795; doi:10.1016/j.epidem.2019.100376)
Supplement: 1 [file NIHMS1578445-supplement-1.pdf]

Phylodynamic analysis of HIV-1 subtypes B, C and CRF 02_AG in Senegal

Fabrícia F. Nascimento[1], Stefan Baral[2], Lily Geidelberg[1], Christinah Mukandavire[1], Sheree R. Schwartz[2], Gnilane Turpin[2], Nguissali Turpin[3], Daouda Diouf[3], Nafissatou Leye Diouf[4], Karleen Coly[2], Coumba Toure Kane[4], Cheikh Ndour[5], Peter Vickerman[6], Marie-Claude Boily[1], and Erik M. Volz[1, 7, *]

[1]Department of Infectious Disease Epidemiology, Imperial College London, Norfolk Place W2 1PG, UK
[2]Department of Epidemiology, Johns Hopkins School of Public Health, Baltimore, MD, USA
[3]Enda Sante, Dakar, Senegal
[4]Institut de Recherche en Santé, de Surveillance Epidemiologique et de Formations, Dakar, Senegal
[5]Division de La Lutte Contre Le Sida et Les IST, Ministry of Health, Dakar, Senegal
[6]Population Health Sciences, Bristol Medical School, University of Bristol, Bristol, UK
[7]MRC Centre for Global Infectious Disease Analysis, Imperial College London, UK
[*]Corresponding author: Erik M. Volz, e.volz@imperial.ac.uk

October 28, 2019

Supplementary Material

Parameter Estimation

We estimated the parameters of our mathematical model using the differential-evolution Markov chain Monte Carlo (MCMC) zs sampler (MCMC-DEzs) (ter Braak and Vrugt, 2008). We initially run few MCMC-DEzs for 3,000 to 4,000 iterations, and we chose one run per analyses to provide initial conditions for subsequent longer runs. Because of computational resources, we run several longer MCMC-DEzs ranging from 10,000 to 15,000 iterations using different initial conditions depending on the analyses. These longer MCMC-DEzs were run in parallel using the computing resources of the Open Science Grid (OSG) (Pordes *et al.*, 2007; Sfiligoi *et al.*, 2009). We merged from 4 to 15 independent runs (also depending on the analyses) in order to have two sets of runs to compare posterior distributions for each parameter and assess convergence of the chains. We also used the Gelman diagnostics to check for convergence.

The calculation of the likelihood used in the MCMC-DEzs was carried out using the function *colik* in R package *phydynR* version 0.1 (Volz, 2017). This function implements the structured coalescent model (SCM) (Volz, 2012) which model each HIV-1 lineage in the phylogeny assuming that each node in the phylogenetic tree corresponds to a single transmission event. For the calculation of

the likelihood we provided a demographic model using the *build.demographic.process* function in *phydynR*, and we set the initial and end time of the calculations to 1978 and 2014, respectively.

For a list of parameters we estimated and their corresponding prior see Table S1.

Table S1: Summary of parameters estimated and MCMC priors

Parameter	Prior
Transmission rate parameter of linear function gp0	Gamma(3, 3/0.1)
Transmission rate parameter of linear function gp1	Gamma(3, 3/0.1)
Transmission rate parameter of linear function gp2	Gamma(3, 3/0.1)
Linear function interval (time) for gp	U(1978, 2014)
Transmission rate parameter of linear function msm0	Gamma(3, 3/0.1)
Transmission rate parameter of linear function msm1	Gamma(3, 3/0.1)
Transmission rate parameter of linear function msm2	Gamma(3, 3/0.1)
Linear function interval (time) for msm	U(1978, 2014)
Risk ratio of *gpm* to transmit to gpf (ψ)	U(0.5, 2)
Importation rate	Exp(30)
Effective population size of *src*	Exp(1/100)
Probability of infected *gpf* to transmit to a *gpm* (p)	Beta(16, 4)
Probability of infected *msm* to transmit to a *msm* (q)	Beta(16, 4)
Initial number of infected *msm*	Exp(1/3)
Initial number of infected *gp*	Exp(1/3)
Removal rate (γ)	Fixed at 1/10

Prevalence and Likelihood Calculation

We computed a statistic to calculate the proportion of infected heterosexual reproductive aged men (*gpm*) that are *msm* that we could compare to our mathematical model. For that we used available HIV-1 prevalence data for *gpm* in 2010 (4.0%, 95% CI: 14% – 80%) and for *msm* in 2016 (29.7%, 95% CI: 21.3% – 38.1%) in Dakar, Senegal (Mukandavire *et al.*, 2018). We also used surveillance data on the proportion of men who are *msm* (1.2%) (Mukandavire *et al.*, 2018), and we assumed that this proportion was independent of the estimated HIV prevalence for *gpm* and *msm*. Using this information, we have:

$$X = q \times p_{msm}/(q \times p_{msm} + (1 - q) \times p_m) \tag{1}$$

Where q is the proportion of males who are *msm*; p_{msm} is HIV prevalence in *msm*; and p_m is HIV prevalence in *gpm*. We also extrapolated and assumed that *msm* HIV prevalence in 2010 was the same as in 2016.

We then approximate the standard deviation of X to a normal distribution, and recomputed X for many replicates of q, p_m and p_{msm} using the differential-evolution Markov chain Monte Carlo zs sampler. We also calculated the “observed” X (X_{OBS}) for 2010 in our phylodynamic analysis, and the mean and standard deviation of X. Using this information we added the term $dnorm(Xobs, Xmean, Xsd, log = TRUE)$ to the calculation of the likelihood. See scripts available at `https://github.com/thednainus/senegalHIVmodel` for further information on how we implemented these calculations in R.

Statistical Analyses

After estimating the parameters of our mathematical model (Table S1), we calculated statistics of interest in molecular epidemiology. We calculated these statistics using the MCMC-DEzs posterior distribution, after removing the burnin, and for maximum a posteriori (MAP) estimates. Note that in SCM as implemented in *phydynR*, we provided the ordinay differential equations (ODEs) as matrices for birth and migration rates, and a vector for the removal rate (Volz, 2012). The birth matrix represents the number of HIV transmissions within the different sub-populations or demes (*gpf*, *gpm*, *msm* and *src*). Similarly, the migration matrix represents allowed transmissions from one sub-population/deme to another sub-population/deme, for example, transmissions from *gpm* to *gpf*.

For each parameter estimates in the posterior distribution, and for the MAP, we solved a demographic model using *phydynR* and estimated the birth and migration matrices, and the effective number of infections for each time point from 1978 to 2014.

In summary, the statistics we calculated for the dynamics of HIV in Senegal were:

- The population attributable fraction (PAF) for each group: *gpf*, *gpm* and *msm*, which are represented by each row in the birth matrix;
- The recent proportion of infections in one group attributable to another group. For example: proportion of infections in *gpf* attributable to *msm*;
- The effective number of infections for each group: *gpm*, *gpf* and *msm*.

Results

Below are the plots for the effective number of infections and population attributable fraction (PAF) for each variation of the model not depicted in the main text.

For the individual analyses for subtype CRF 02_AG and subtype C, the following applies:

- Model 1: We assigned each sequence to its respective risk-group in the phylogenetic tree a value of 1.0 (100% in the respective self-reported risk group);
- Model 2: We assumed some uncertainty in the self-reported *gpm* by assigning to every *gpm* sequence a value of 0.5 (50%) of being *gpm* and 0.5 (50%) of being *msm*;
- Model 3: We assigned each sequence to its respective risk-group in the phylogenetic tree a value of 1.0 (100% in the respective self-reported risk group) and added the prevalence term to the calculation of the likelihood. This plot is only shown in the main text only;
- Model 4: We assumed some uncertainty in the self-reported *gpm* by assigning to every *gpm* sequence a value of 0.5 (50%) of being *gpm* and 0.5 (50%) of being *msm*. We also added the prevalence term to the calculation of the likelihood.

For the combined analyses using data from subtypes B, C and CRF 02_AG the following applies:

- Model 1: We assigned each sequence to its respective risk-group in the phylogenetic tree a value of 1.0 (100% in the respective self-reported risk group);
- Model 2: We removed all *gpm* sequences from the phylogenetic tree;
- Model 3: We assumed some uncertainty in the self-reported *gpm* by assigning to every *gpm* sequence a value of 0.5 (50%) of being *gpm* and 0.5 (50%) of being *msm*;

- Model 4: We assigned each sequence to its respective risk-group in the phylogenetic tree a value of 1.0 (100% in the respective self-reported risk group) and added the prevalence term to the calculation of the likelihood. This plot is only shown in the main text only;

- Model 5: We removed all *gpm* sequences from the phylogenetic tree and added the prevalence term to the calculation of the likelihood;

- Model 6: We assumed some uncertainty in the self-reported *gpm* by assigning to every *gpm* sequence a value of 0.5 (50%) of being *gpm* and 0.5 (50%) of being *msm* and added the prevalence term to the calculation of the likelihood.

Subtype C: Model 2

For subtype C model 2 we noticed that the MCMC runs for three parameters representing the linear function for *msm* did not converge to the same posterior distribution. This could be attributable to non-identifiability of these parameters. For MCMC posterior probability plots, see `analyses/results/plots/mcmc_runs` within the GitHub repository for the Senegal analyses. `https://github.com/thednainus/senegalHIVmodel`.

To better understand if this was a potential non-identifiability problem, we solved PAF and effective number of infections using both results from the MCMC posterior distributions. Results were very similar as observed at `results/plots/plots_withMaleX/subtypeC_model2` in the GitHub repository `https://github.com/thednainus/senegalHIVmodel`.

Effective Number of Infections

Figure S1: **Effective number of infections for subtype CRF 02_AG.** Plots showing the proportion of the effective number of infections for *gpf*, *gpm* and *msm*. MAP = maximum a posteriori.

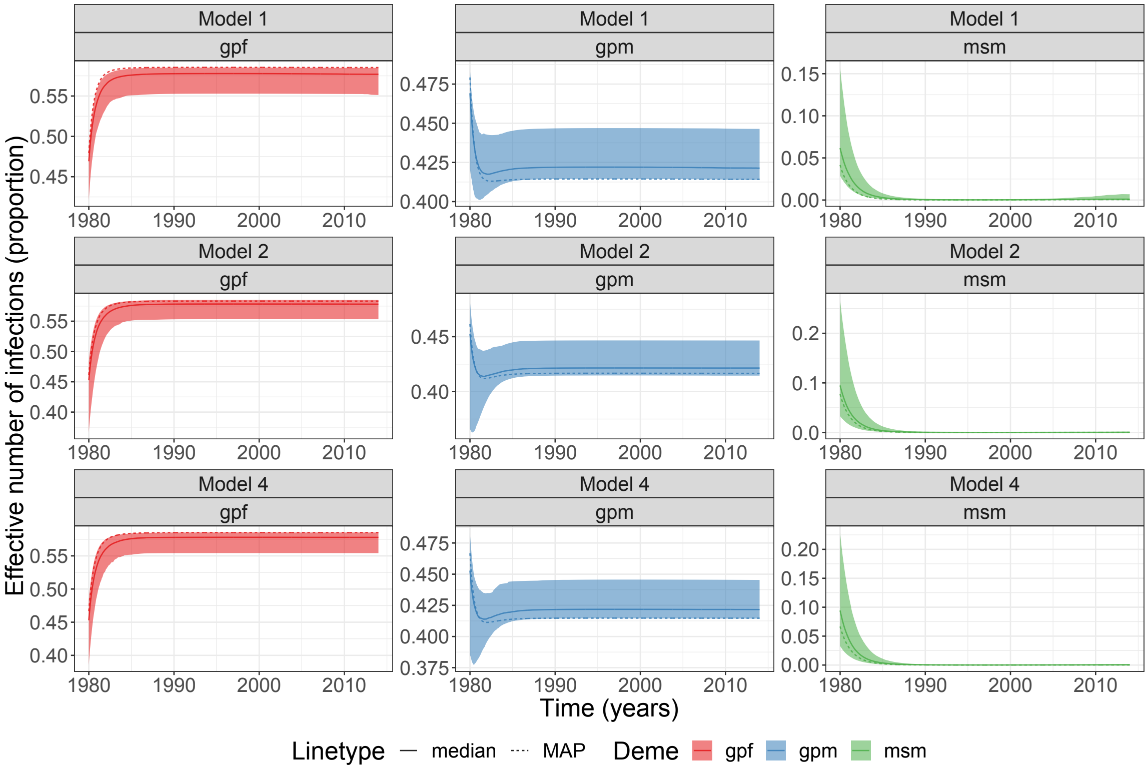

Figure S2: **Effective number of infections for subtype C.** Plots showing the proportion of the effective number of infections for *gpf*, *gpm* and *msm*. MAP = maximum a posteriori.

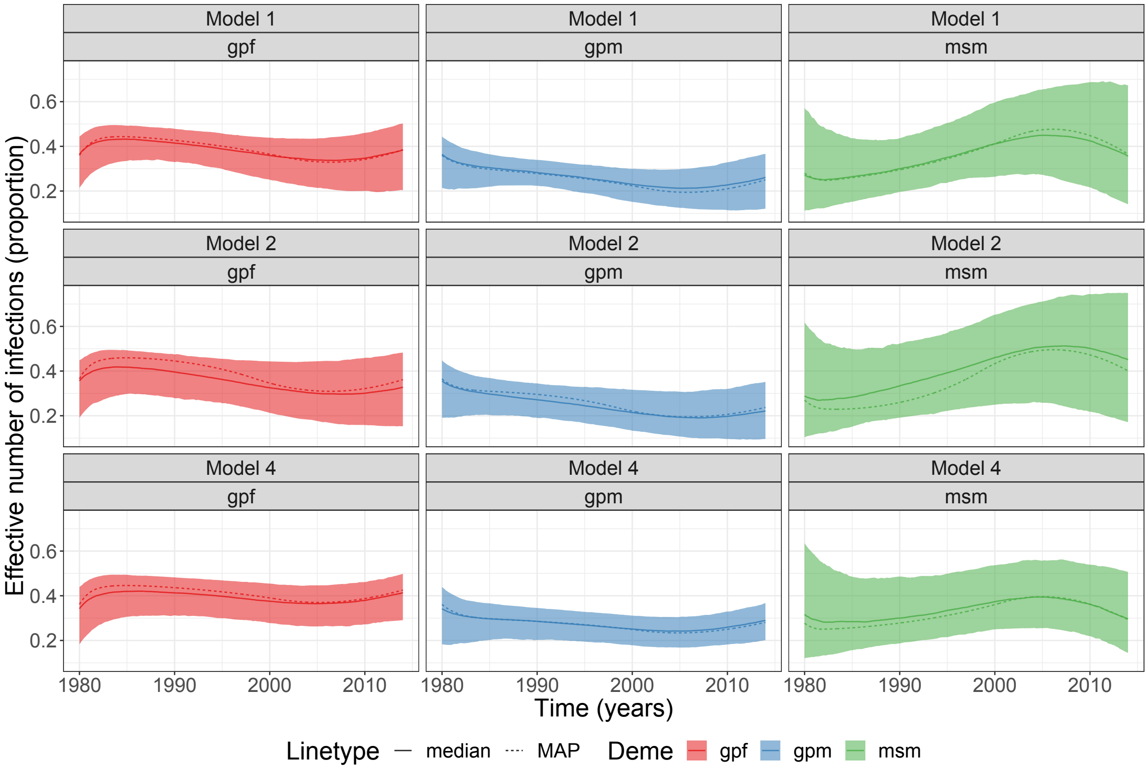

Figure S3: **Effective number of infections for combined analyses.** Plots showing the proportion of the effective number of infections for *gpf*, *gpm* and *msm*. MAP = maximum a posteriori.

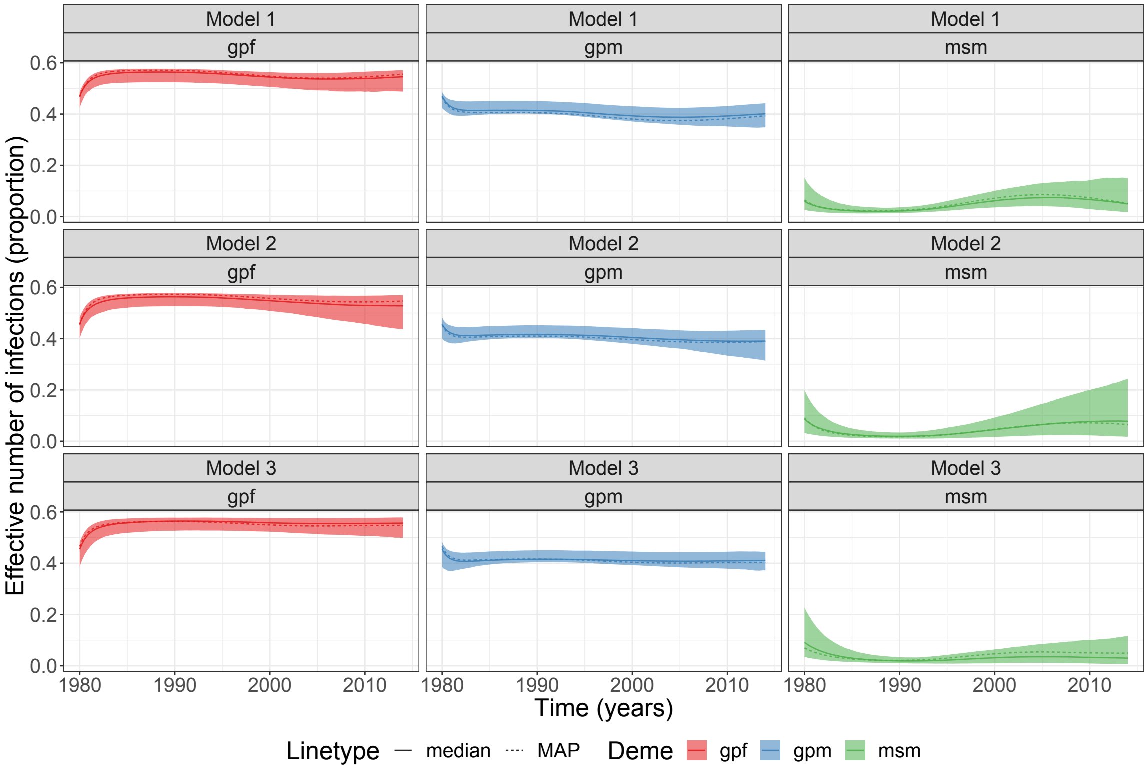

Figure S4: **Effective number of infections for combined analyses.** Plots showing the proportion of the effective number of infections for *gpf*, *gpm* and *msm*. MAP = maximum a posteriori.

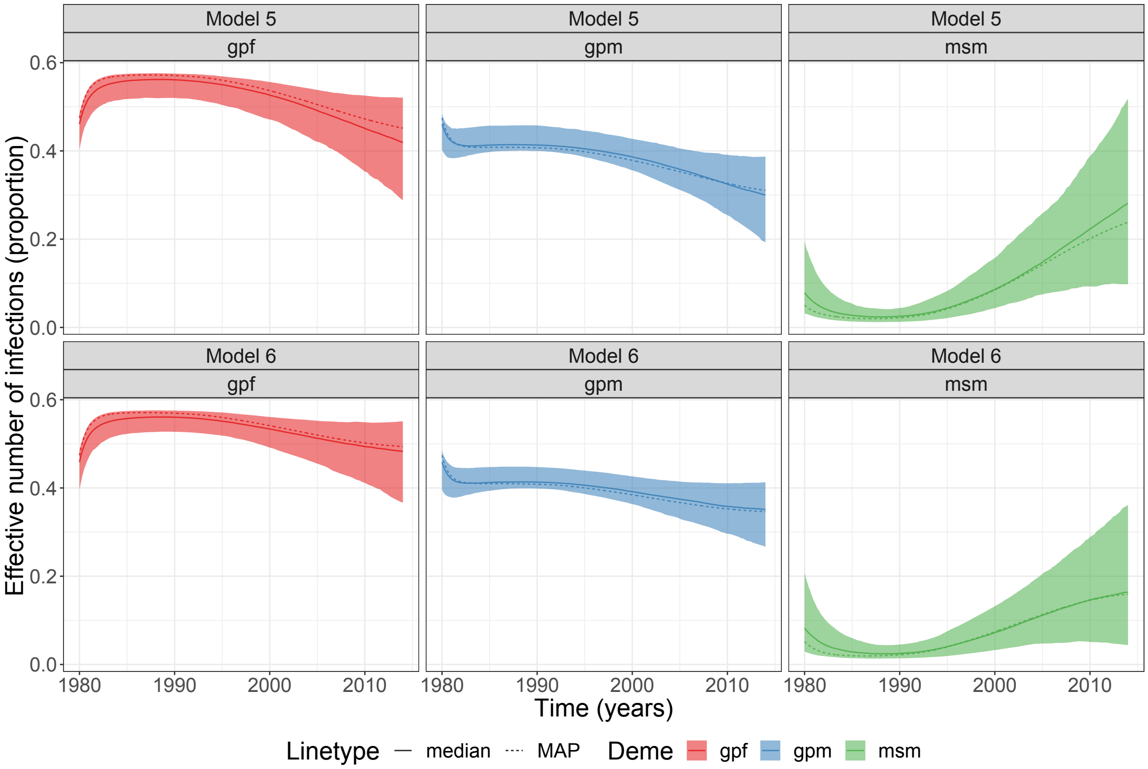

Population attributable fraction

Figure S5: **Population attributable fraction for subtype CRF 02_AG.** Plots showing the population attributable fraction for *gpf*, *gpm* and *msm*. Point estimates and error bars in the last plot represents 1-year PAF estimated for MSM in Mukandavire *et al.* (2018). MAP = maximum a posteriori.

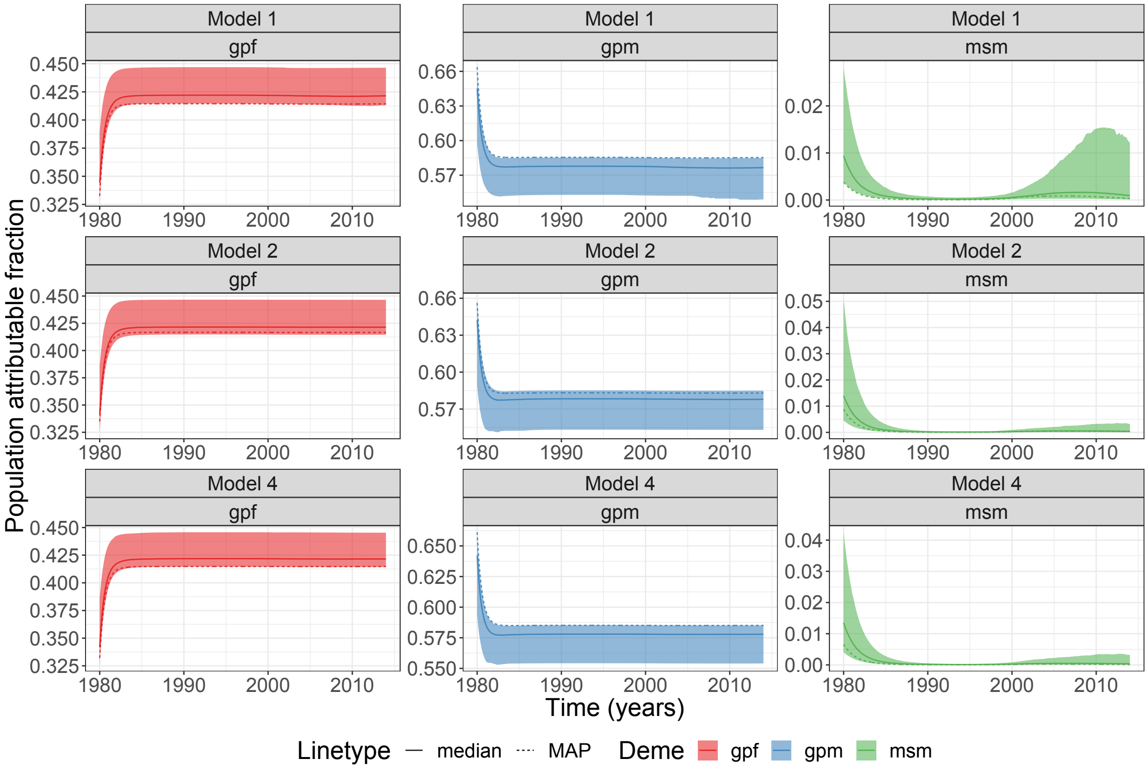

Figure S6: **Population attributable fraction for subtype C.** Plots showing the population attributable fraction for *gpf*, *gpm* and *msm*. Point estimates and error bars in the last plot represents 1-year PAF estimated for MSM in Mukandavire *et al.* (2018). MAP = maximum a posteriori.

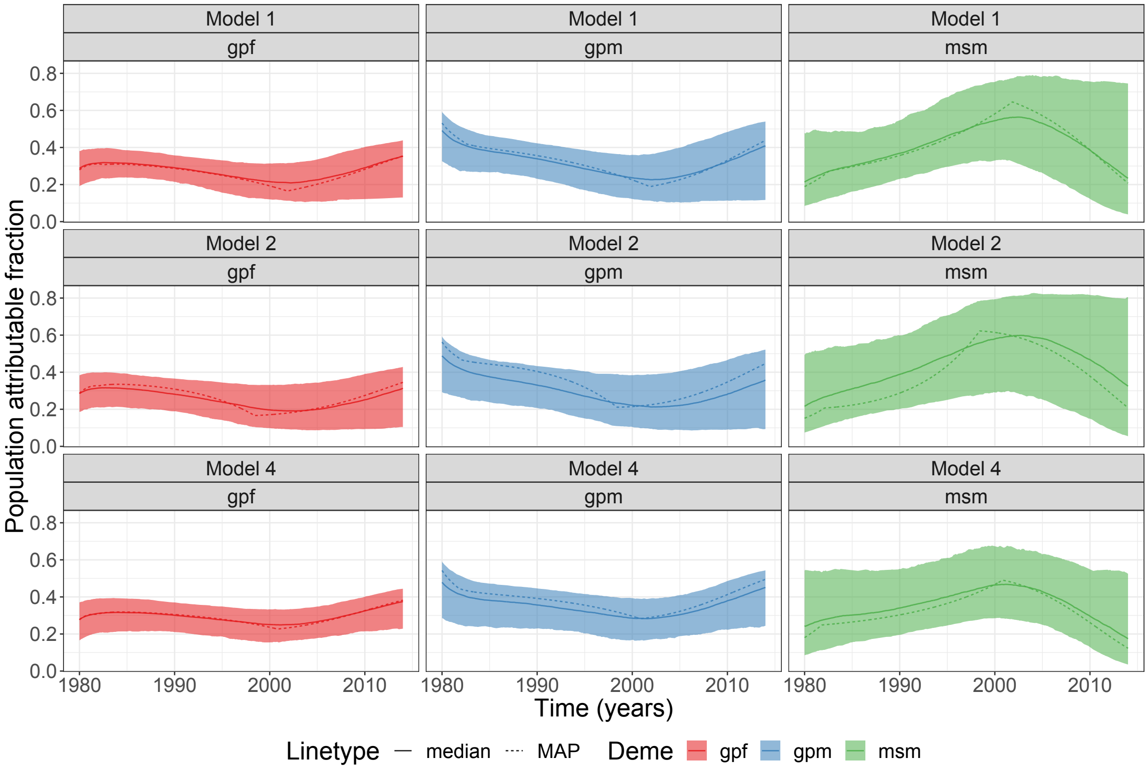

Figure S7: **Population attributable fraction for the combined analyses.** Plots showing the population attributable fraction for *gpf*, *gpm* and *msm*. Point estimates and error bars in the last plot represents 1-year PAF estimated for MSM in Mukandavire *et al.* (2018). MAP = maximum a posteriori.

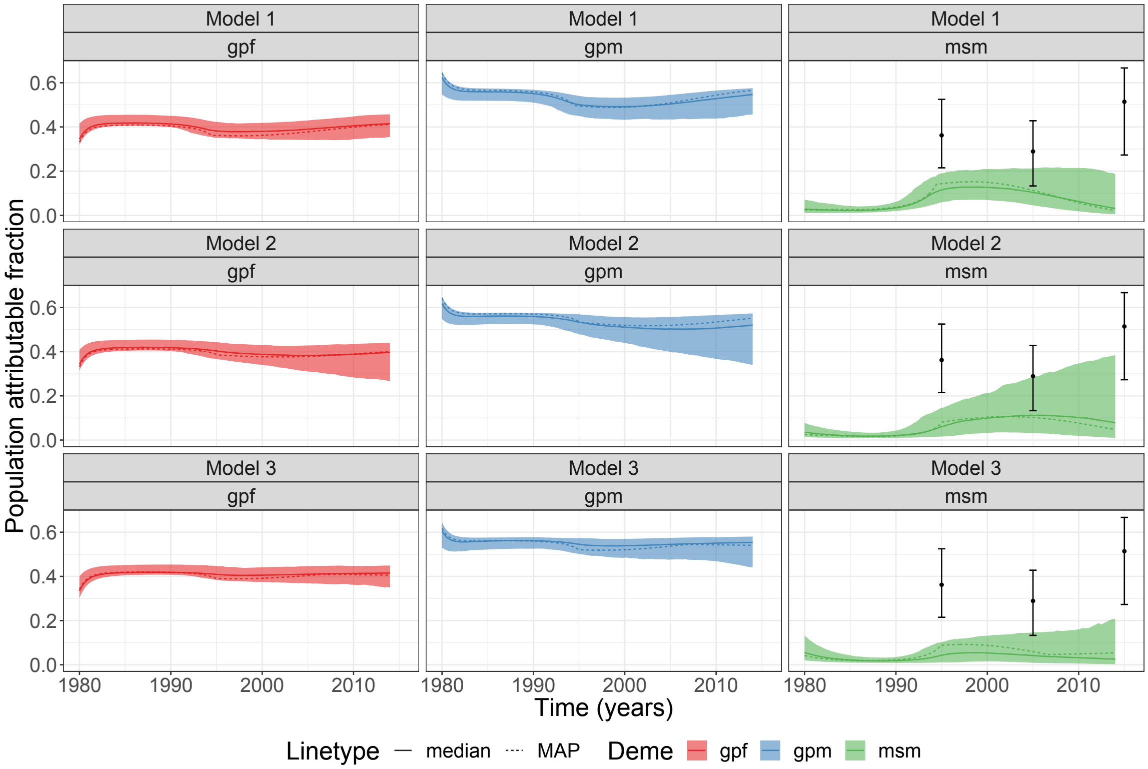

Figure S8: **Population attributable fraction for the combined analyses.** Plots showing the population attributable fraction for *gpf*, *gpm* and *msm*. Point estimates and error bars in the last plot represents 1-year PAF estimated for MSM in Mukandavire *et al.* (2018). MAP = maximum a posteriori.

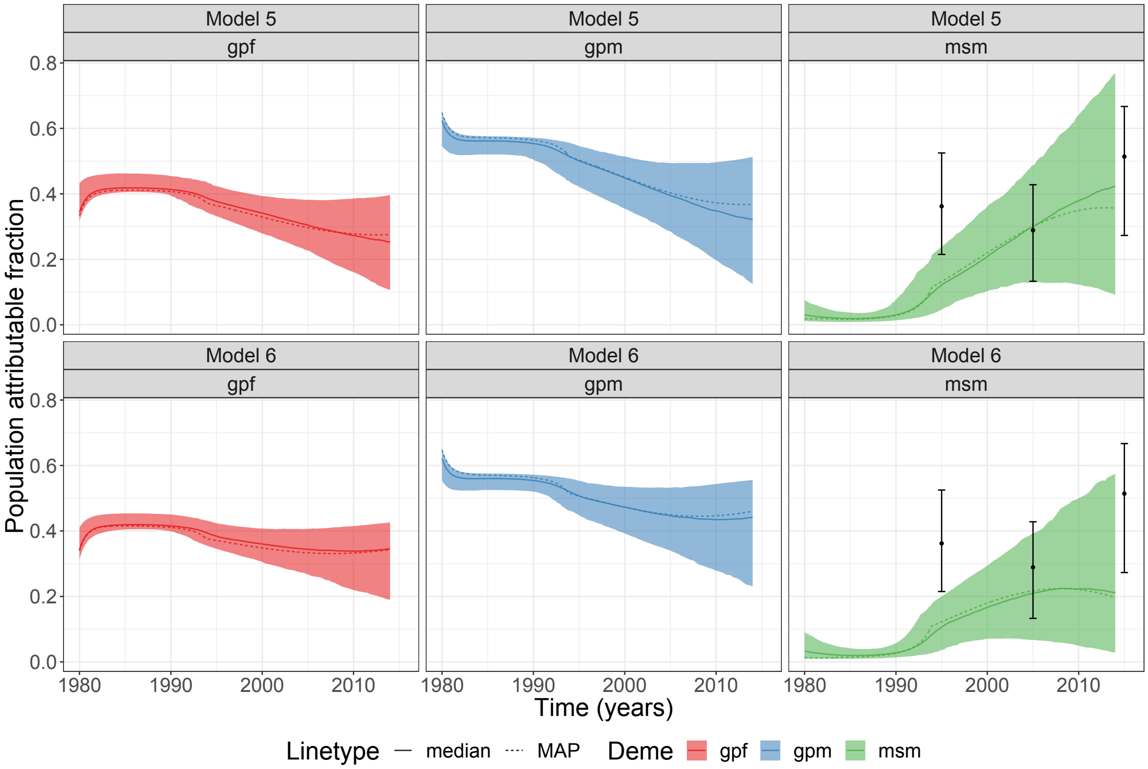

References

Mukandavire, C., Walker, J., Schwartz, S., Boily, M., Danon, L., Lyons, C., Diouf, D., Liestman, B., Diouf, N. L., Drame, F., Coly, K., Muhire, R. S. M., Thiam, S., Diallo, P. A. N., Kane, C. T., Ndour, C., Volz, E., Mishra, S., Baral, S., and Vickerman, P. (2018). Estimating the contribution of key populations towards the spread of HIV in Dakar, Senegal. *Journal of the International AIDS Society*, **21**(Suppl Suppl 5).

Pordes, R., Petravick, D., Kramer, B., Olson, D., Livny, M., Roy, A., Avery, P., Blackburn, K., Wenaus, T., Würthwein, F., Foster, I., Gardner, R., Wilde, M., Blatecky, A., McGee, J., and Quick, R. (2007). The open science grid. In *Journal of Physics: Conference Series*, volume 78.

Sfiligoi, I., Bradley, D. C., Holzman, B., Mhashilkar, P., Padhi, S., and Würthwein, F. (2009). The pilot way to Grid resources using glideinWMS. In *2009 WRI World Congress on Computer Science and Information Engineering, CSIE 2009*, volume 2, pages 428–432.

ter Braak, C. J. F. and Vrugt, J. A. (2008). Differential Evolution Markov Chain with snooker updater and fewer chains. *Statistics and Computing*, **18**(4), 435–446.

Volz, E. (2017). phydynR: Phylogenetic dating and phylodynamic inference by sequential Monte Carlo. `https://github.com/emvolz-phylodynamics/phydynR`, visited 2019-01-22.

Volz, E. M. (2012). Complex population dynamics and the coalescent under neutrality. *Genetics*, **190**(1), 187–201.
